# A scalable approach to topographically mediated antimicrobial surfaces based on diamond

**DOI:** 10.1186/s12951-021-01218-3

**Published:** 2021-12-28

**Authors:** William F. Paxton, Jesse L. Rozsa, Morgan M. Brooks, Mark P. Running, David J. Schultz, Jacek B. Jasinski, Hyun Jin Jung, Muhammad Zain Akram

**Affiliations:** 1 Conn Center for Renewable Energy Research, University of Louisville, Louisville, KY 40292 USA; 2 219 Life Sciences Building, University of Louisville, Louisville, KY 40292 USA; 3 LSU School of Medicine, 1542 Tulane Ave, New Orleans, LA 70112 USA; 4 Kentucky Advanced Materials Manufacturing, Louisville, KY 40209 USA

**Keywords:** Topographically mediated surfaces, Diamond, Nanoneedles, Nanospikes, Antimicrobial, CVD

## Abstract

Bio-inspired Topographically Mediated Surfaces (TMSs) based on high aspect ratio nanostructures have recently been attracting significant attention due to their pronounced antimicrobial properties by mechanically disrupting cellular processes. However, scalability of such surfaces is often greatly limited, as most of them rely on micro/nanoscale fabrication techniques. In this report, a cost-effective, scalable, and versatile approach of utilizing diamond nanotechnology for producing TMSs, and using them for limiting the spread of emerging infectious diseases, is introduced. Specifically, diamond-based nanostructured coatings are synthesized in a single-step fabrication process with a densely packed, needle- or spike-like morphology. The antimicrobial proprieties of the diamond nanospike surface are qualitatively and quantitatively analyzed and compared to other surfaces including copper, silicon, and even other diamond surfaces without the nanostructuring. This surface is found to have superior biocidal activity, which is confirmed via scanning electron microscopy images showing definite and widespread destruction of *E. coli* cells on the diamond nanospike surface. Consistent antimicrobial behavior is also observed on a sample prepared seven years prior to testing date.

**Supplementary Information:**

The online version contains supplementary material available at 10.1186/s12951-021-01218-3.

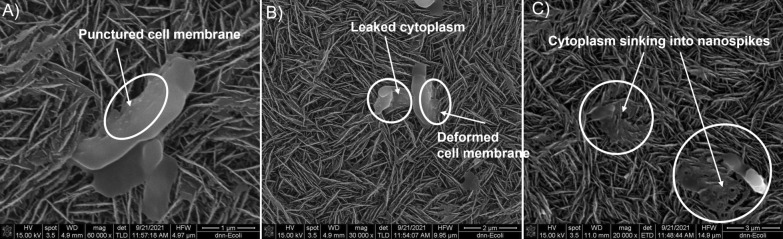

## Introduction

The spread of infectious diseases is inarguably one of the biggest threats to civilization, a fact highly validated by the COVID-19 pandemic caused by the SARS-CoV-2 virus. Emerging Infectious Disease (EID) events have unfortunately been on the rise; thus the probability of another pandemic will continue to increase unless we develop novel means to prevent the spread of such contagions [1, 2]. EIDs can be classified into four main types, bacterial, viral, parasitic, and fungal, accounting for 54%, 25%, 14%, and 3%, respectively of 335 EIDs studied between 1940 and 2004 [1]. The high percentage of bacterial-related EIDs should be especially concerning when noting the increasing prevalence of antibiotic-resistance bacteria strains and a pandemic caused by such could dwarf the fatalities caused by SARS-CoV-2. Infectious pathogens are typically removed from surfaces by disinfectant sprays/wipes, ultra-violet light exposure, and/or heat, among others. While such techniques have proven effective when properly conducted, they must be frequently executed and are subject to human error, e.g., neglected spots and missed cleaning intervals. Clearly, the most effective solution would be an active surface with long-lasting antimicrobial properties.

Certain materials such as copper exhibit inherent antimicrobial properties but typically lose efficacy over long periods of time, as they undergo oxidation through interactions with cellular molecules or simply from exposure to air [3]. Attempts to invoke antimicrobial properties on common materials such as stainless steel include depositing biocidal metals, biopolymers, inorganic oxides, and N-halmines [4–6]. Few if any of these techniques have been successfully commercialized due to high costs, lack of versatility, and a requirement to “recharge” the surface after some period of time to maintain their antimicrobial properties.

Recent research has focused on the development of surfaces that maintain their antimicrobial properties over exceptionally long time periods. One promising technique involves the use of micro/nano-scale topographical features to control cell interactions. These Topographically Mediated Surfaces (TMSs) may be thought of as a form as biomimicry, as early attempts were highly influenced by the cicada wing’s biocidal properties which is attributed solely to surface structure [7]. A clever evolutionary result for rapid adaptation, the cicada wing is comprised of chitin, protein, and wax covered with nanopillars that have been shown to kill *Pseudomonas aeruginosa* within three minutes of contact [7–9]. The effectiveness of densely-packed nanopillars is believed to be due to a ‘bed of nails’ effect whereby the bacterial cell walls are elongated/deformed and/or punctured, killing them on contact. A similar theory attributes the biocidal effect to a combination of adhesion between the nanopillars and the bacterium membrane along with the shear forces caused when a bacterium attempts to relocate from a nanopillar, resulting in “ripping” of the cell membrane [10, 11].

Several bio-mimicking, antimicrobial TMSs have been developed and tested over the past decade. Black silicon was one of the first such surfaces explored due to its similarity to a cicada wing and well-documented fabrication process. Black silicon was indeed confirmed to demonstrate highly effective biocidal properties against Gram-negative and Gram-positive bacteria, along with endospores with exceptionally high killing rates [12]. Several follow-up studies have created similar structures out of various materials such as polymers (including: polyethylene terephthalate and polymethyl methacrylate), metals (including: titanium, gold, and aluminum), and ceramics/semiconductors (including: titania and diamond, which is explored in the current study) [13–19]. A common shortfall of all known previously reported techniques is their reliance on costly microfabrication processes such reactive ion beam etching, lithographic patterning, and focused ion beam milling, all of which cannot scale for mass production of coatings larger than ~ 1 m^2^. The present study details an as-deposited, diamond-based antimicrobial surface, which is the first known to be able to demonstrate the desired biological properties as those previously mentioned, while not requiring any post processing.

The use of diamond for antimicrobial applications is not new, with interest driven by its many superior material properties such as chemical inertness, hardness, corrosion resistance, and ability to deposit with countless morphologies. Diamond and diamond-like carbon (amorphous carbon primarily comprised of sp^3^ bonds) has also been well documented to be extremely biocompatible [20–22]. For instance, it has been reported the chemical vapor deposited (CVD) diamond films are as biocompatible as titanium and 316 stainless steel, but with less in vitro and in vivo cellular adhesion and activation [22]. Further, methods to deposit diamond have drastically improved over the past 10 + years, enabling large-area deposition of thin films at relatively low costs of well under $100/m^2^ [23, 24]. Several past studies exploring diamond in this area focus on surface functionalization as means to invoke antimicrobial behavior. Work has shown positive results from nanoparticles and surfaces with functional groups including amines (NH_2_), mannose, fluorine, and even menthol [25–27]. Other diamond antimicrobial studies have found untreated nanodiamond to also be unfavorable to bacterial growth, which has been attributed to surface hydrophobicity, low surface energy and roughness [28], and bacterial cell binding and subsequent cellular disruption in the case of nanoparticles [28–30].

More recent studies have sought to combine the beneficial effects of TMSs and diamond into one surface with some success. Creating sharp needle- or spike-like structures seen in typical TMS surfaces is more difficult with diamond micro/nano fabrication processes compared to silicon. Accordingly, several studies have simplified the processes for characterization by simply coating pre-fabricated black silicon surfaces with diamond, resulting in a diamond needle-like surface with a larger radius of curvature on each tip than the base black silicon [31]. Despite the previously mentioned advantageous properties of nanodiamond, the use of it as a coating on black silicon only resulted in marginal improvements over standard black silicon, likely due to the decreased aspect ratios and feature spacing [19, 31, 32].

It is clear that TMSs demonstrate superior antimicrobial performance and appear to be agnostic to the base material, as cellular disruption is primarily attributed to mechanical interaction. Large-scale implementation of such surfaces using previously reported techniques would be extremely difficult, as they all rely on micro/nano fabrication techniques that would be unsuitable for exceptionally large surfaces such as tables, flooring, ventilation components, etc. The present study explores an alternative and promising approach to creating a TMS utilizing diamond without requiring any post-processing and could easily be scaled to invoke antimicrobial properties on a wide range of high-touch surfaces. By precisely controlling the deposition parameters, a nano-diamond surface can obtain a morphology similar to a ‘bed of nails’, which is typical of almost all TMS demonstrations. Antimicrobial behavior of several diamond nanospike (DNS) surfaces was characterized and compared against standard materials.

DNS surfaces were synthesized via microwave plasma-enhanced chemical vapor deposition with methane, hydrogen, and nitrogen as feed gases. A more detailed description of the synthesis procedure is presented in Additional file 1. Diamond coatings with high aspect ratio features have been explored in previous studies for electrical/electrochemical electrodes to increase the active surface area [33]. The use of high levels of nitrogen, along with precise control of other deposition parameters, has been shown to enable directionally preferential growth of grains from nucleation sites. Such control allows enhanced growth rates along specific longitudinal directions compared with circumferential growth [34].

It should be further emphasized here that all testing was performed on as-deposited samples. No specific pre-treatment or post-deposition fabrication methods were required. Such ability places these diamond structures at a distinct advantage over all other known TMSs, which require extensive microfabrication processes.

**Fig. 1:**
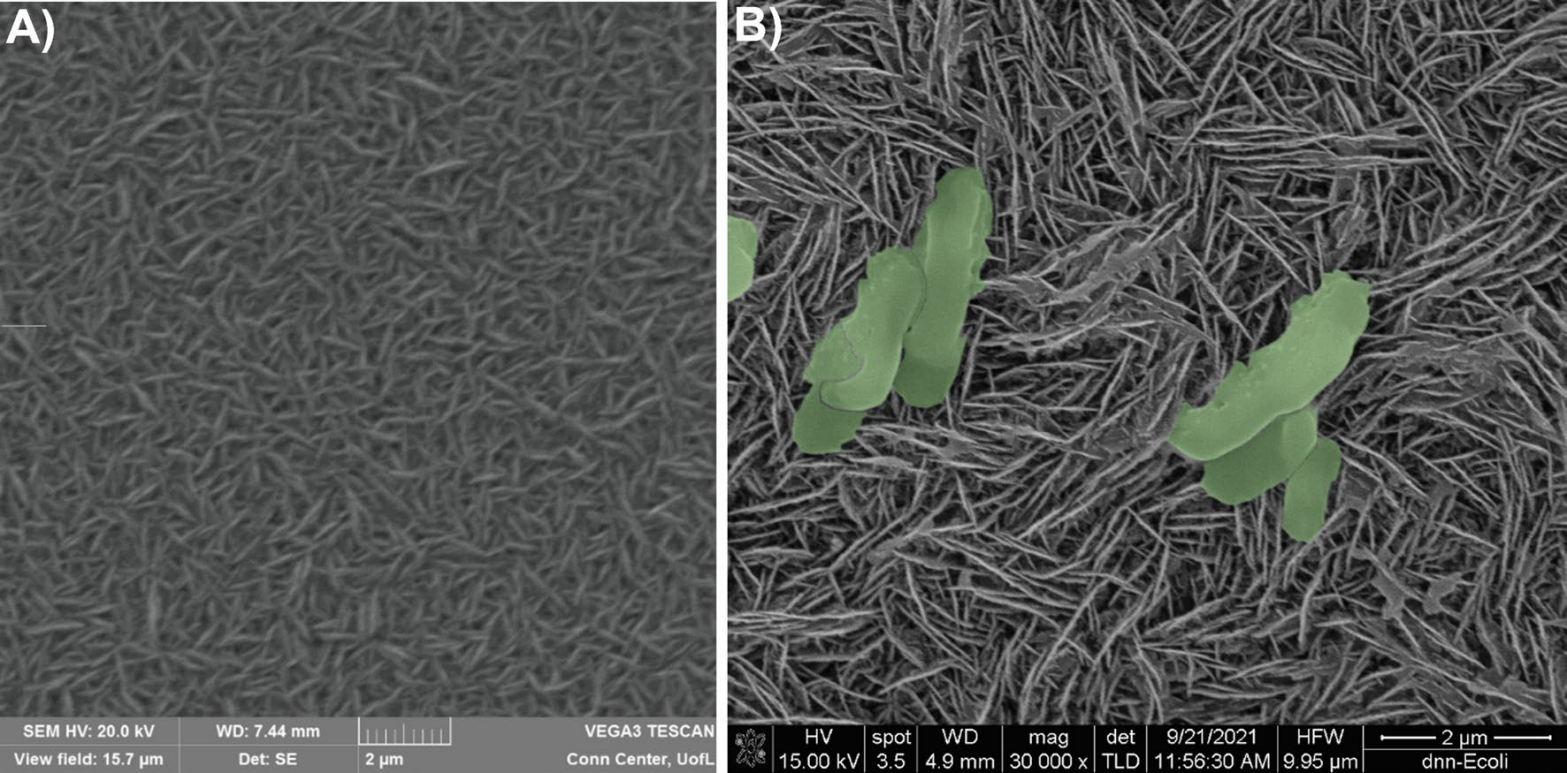
**A** SEM image of the as-deposited DNS structures at 20k× magnification. Densely packed, sharp needle- or spike-like structures can be easily discerned with heights of around 1 µm and widths of ~ 50 nm. **B** SEM image of *E. coli* cells severely disrupted by the diamond nanospikes

The resulting films were extremely black in appearance upon initial inspection, a characteristic property of black silicon, indicating pronounced light absorption from the nanoscale features [35]. It can be seen from the scanning electron microscopy (SEM) image in Fig. 1A that the surface morphology is comprised of a highly dense, randomly oriented array of high aspect ratio, sharp needle-or spike-like structures with heights and widths around 1 µm and 50 nm, respectively. A cross sectional SEM image of a tested film is presented in Additional file 1: Fig. S1A. The height of 10 µm corresponds to a growth of around 1um/hr. AFM mapping results for DNS film are shown in Additional file 1: Fig. S1B. The root mean square (RMS) surface roughness value of 63.899 nm was measured by AFM. Raman spectroscopy (Additional file 1: Fig. S2) of the films was also performed, which confirmed the surface is primarily diamond with a minority non-diamond, sp^2^ contribution [36].

An initial series of biological testing was performed to qualitatively assess the antimicrobial properties of this DNS surface. Moreover, these initial tests were performed on a sample (Sample A) synthesized 7 years prior to examination, which provides insight into the efficacy of such surfaces years after initial installation under typical conditions. Sample A had been kept in storage in a plastic petri dish with a lid at standard atmospheric conditions with no specific care taken. This initial testing consisted of simply comparing the organismal activity on the DNS surface with five common materials found on commercially available surfaces: 316 stainless steel, galvanized steel, polyethylene plastic, and copper.

A series of collections was taken on the above surfaces along with both positive and negative controls. Collections were taken first at Day 0 to confirm no prior contamination before starting the experiment. Each sample, except the negative control, was then exposed to a known bacterial source consisting of both a nasal and skin swab of a healthcare worker after a shift in a general medical ward. Post-exposure collections were taken at 48 h (Day 2) and 96 h (Day 4), and spread across Lysogeny broth agar plates which were then cultured for five days (120 h). A more detailed description of experimental procedures is presented in the Additional file 1.

**Fig. 2:**
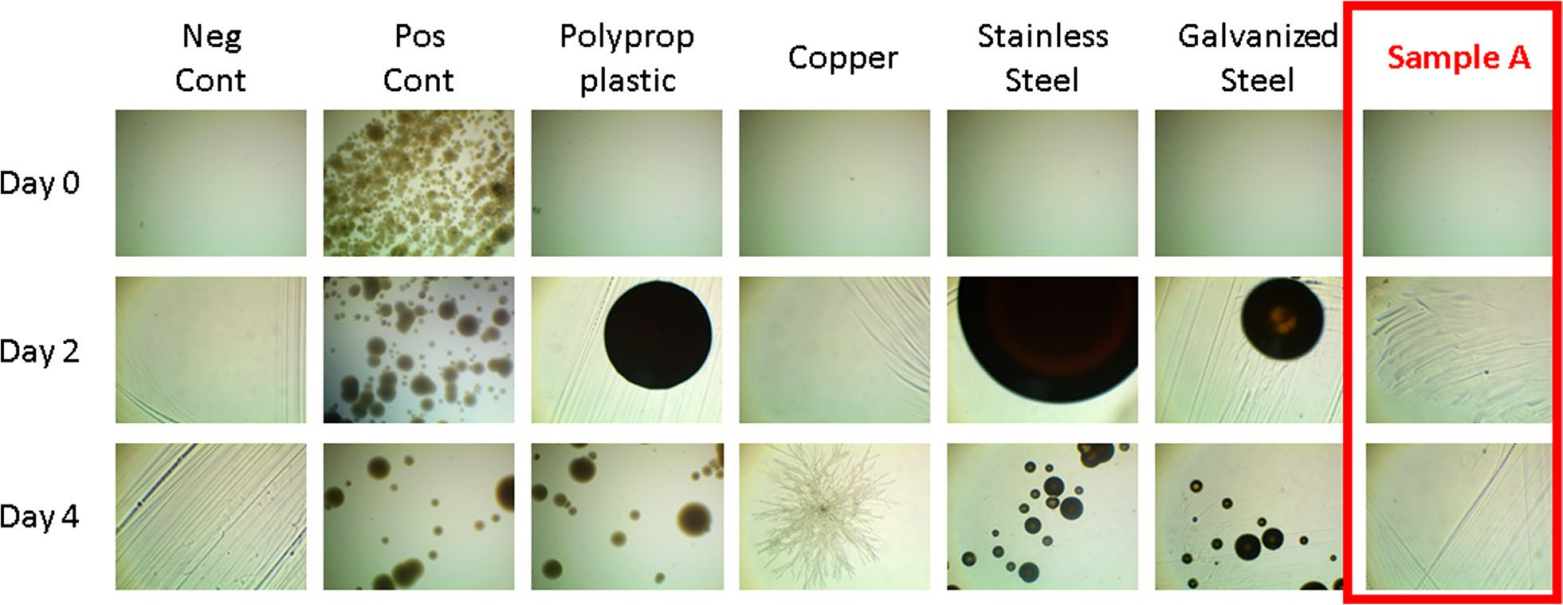
Bacterial growth observations of a negative control (no bacteria exposure), positive control, polypropylene plastic, galvanized steel, stainless steel, copper, and DNS coating (Sample A). Collections were taken on each day of the table and the images are the resulting growth after collections cultured for 5 days. Day 0 had no exposure to pathogens

Observation of Fig. 2 shows no observable growth on the negative control, confirming all materials used had no contamination prior to testing. The positive control had pronounced microbial growth which verified the positive source indeed contained bacteria. Polypropylene plastic, galvanized steel, stainless steel, and copper all showed some microbial growth whereas the diamond nanospikes showed no observable microbial growth. Interestingly, copper did not appear to have any bacterial growth (as to be expected), however it did show indications of fungal growth. Previous studies appear to confirm this possibility and have shown that while copper is predominately antifungal, certain fungi strains such as *Aspergillus Niger* demonstrate a pronounced resilience to copper [37].

A second set of studies was performed on Sample A to further qualitatively test long-term efficacy of antimicrobial properties arising from the DNS surface. For this study a single collection for each surface was obtained (using the same technique as above) 28 days post-exposure to the positive source. This roughly one-month time data point was chosen because any viable microorganisms should certainly demonstrate some growth after this amount of time. Pictures were then taken at the time of collection (0 h) and every 24 h for a maximum of 120 h.

A table of macroscopic images of the petri dishes onto which the collections were smeared can be seen in Additional file 1: Fig. S5. Similar results to Fig. 2 were found whereby the negative control had no growth while the positive control and all other surfaces had observable microbial growth except for the DNS surface It should be reiterated that these first two ‘qualitative’ tests were initially performed only to provide preliminary indication into the antimicrobial properties of the reported diamond-based surface. These studies have been included because the results clearly indicate that the diamond structures appear to follow other TMSs and exhibit a clear antimicrobial effect, specifically in real-world scenarios, as the organismal collections were taken from a healthcare worker exposed to the everyday environment of a large public hospital. Additionally, this study should be of particular interest to all TMS-related research as it characterized a surface synthesized seven years prior. This is the first known report of the long-term efficacy of a TMS and strongly indicates the potential of such surfaces to reduce the spread of future emerging infectious diseases.

Additional follow-up biological characterization was performed on several additional samples deposited within one month of testing and compared against common materials. These experiments were focused on quantifying the antibacterial behavior using *E. coli*, a gram-negative bacterium frequently used in previously reported work studying similar TMSs [8, 10, 11]. The *E. coli* was cultured and aliquoted in equal amounts on a sample of copper, silicon, polycrystalline diamond, and DNS surfaces along with a positive control.

The cultures were allowed to dry in a Laminar flow hood for 15 min. The samples and controls with aliquots of *E. coli* were incubated at 37 °C for 24 h, after which 1 mL of sterile lysogeny broth media was used to harvest the *E. coli* off the samples. These samples were then loaded onto an optical density plate and absorption measurements were taken at a wavelength of 600 nm every 30 min [30].

The polycrystalline diamond tested here was an as-deposited, freestanding sample with a crystal morphology comprising large grains of > 10 µm with wide bases, rounded tops, and low aspect ratios. While this sample is comprised of diamond, it has vastly different morphology compared to the diamond nanospikes of interest to the present study and was included to determine whether antimicrobial properties were due to the diamond or to the nanostructured surface. Similarly, silicon was chosen because it was the substrate for the DNS surface in this study and it was desired to eliminate any contribution of the silicon to overserved biological properties.

**Fig. 3:**
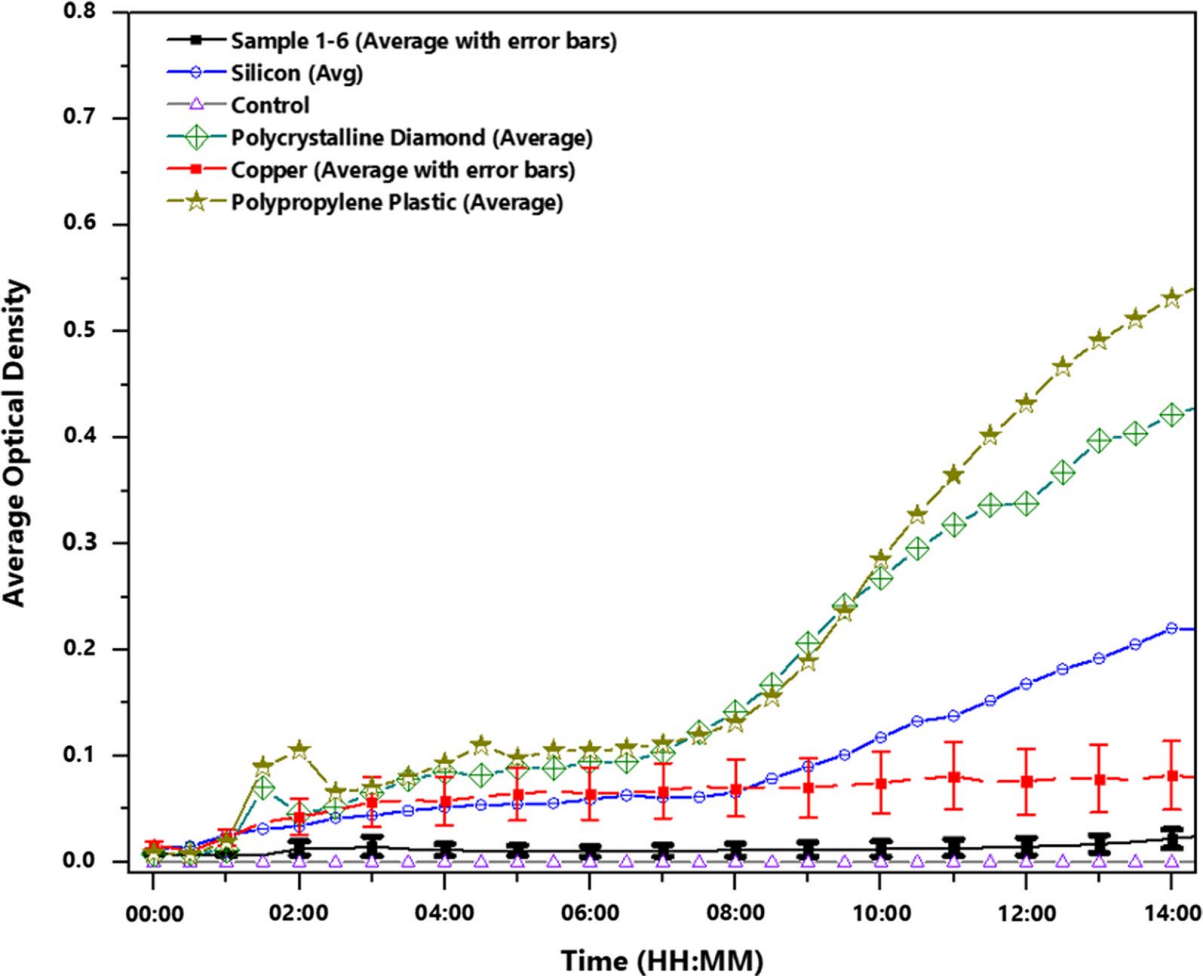
Average optical density curves of DNS surfaces (6 samples of 11 total trials), copper (5 trials), silicon (3 trials), polycrystalline diamond (3 trials), and a control (3 trials). Increased optical density directly correlates with bacterial growth on samples

Figure 3 provides strong evidence for the antibacterial properties of the DNS surface. The negative control consistently showed no growth above the background, confirming the validity of this testing technique. Both the silicon and the microcrystalline diamond surfaces had pronounced growth of *E. coli*, which eliminates both the diamond itself and the silicon substrate as contributing to the antimicrobial properties seen from the DNS surface.

Further validation of the diamond nanospike’s antimicrobial properties employed SEM to observe interaction of *E. coli* cells with the structured surface. *Escherichia coli *was exposed to both a control silicon and a DNS surface in the same manner as previously discussed for the qualitative testing. Samples were then dried, sputtered with gold and placed in the SEM. Figure 1B shows the DNS surface at 30k× magnification with the *E. coli* cells artificially colorized in green for easier visualization. The *E. coli* cells are clearly damaged by the DNS surface consistent with the ‘ripping’ model previously discussed [10, 11]. Additional images and a description of the stages of cell destruction can be found in the Additional file 1 along with an image of healthy *E. coli* on a control silicon substrate for comparison (Additional file 1: Figs. S3 and S4). These SEM results present definitive proof that the surface is not only antimicrobial but biocidal in that it destroys bacteria on contact.

TMSs have been explored in much detail using a variety of different materials. Further, past studies have fabricated diamond-based TMSs, primarily depositing on black silicon. The antimicrobial/antibacterial properties of the DNS surfaces observed in the present study accordingly, are to be expected as the morphology demonstrates sharp, high aspect ratio structures in similar size and density to those previously explored. The fundamental difference between these structures and those previously reported is that the diamond nanospikes were all in their as-deposited state with no subsequent micro/nanofabrication processes employed (such as lithography, ion etching, etc.). This fundamental difference (and prime novelty) overcomes the major hurdle for widespread implementation of TMSs to prevent other EIDs by dramatically reducing fabrication costs. Additionally, this study is the first known to characterize the antimicrobial properties of a TMS seven years after initial synthesis. These results provide strong, positive evidence into the long-term efficacy of any TMS comprised of a stable material, resistant to atmospheric effects such as oxidation.

To fully capitalize on any TMS, large-scale deposition is required. Not only do the diamond-based surfaces in the present study eliminate the need for unscalable, costly post-processing but can also benefit from the recent advancements in diamond reactor technologies. Several techniques have been developed for depositing diamond, especially diamond surfaces with nanocrystalline morphologies. Target markets driving this development include electrochemical electrodes and sensors along with wear resistant, optically transparent, and/or chemically resistant coatings. Reactor technologies are consistently being developed with the ability to coat larger and larger sizes with commercially available hot filament chemical vapour deposition (CVD) and linear array microwave plasma CVD systems able to deposit areas of square meters on a wide range of surfaces [38, 39]. Further, nanodiamond films similar to the DNS structures explored in the present study have been successfully deposited on a wide range of materials. Ideal substrates for such deposition are materials which readily form carbides such as silicon, molybdenum, tungsten, etc. Recent success as vastly expanded this list to include non-carbide forming materials such as stainless steel, copper, and quartz using a variety of preparation techniques and interface layers [40–42].

## Conclusions

In conclusion, a diamond-based antimicrobial surface has been synthesized with antimicrobial properties comparable to copper. This surface can be classified as a topographically mediated surface in that it utilizes sharp, high aspect ratio nanostructures to mechanically disrupt bacteria on contact and prevent further growth. This technique is the first known to accomplish such a surface without the use of expensive micro/nanofabrication processes which are not scalable. Using current diamond fabrication techniques, this coating can be sufficiently scaled to address many applications involving high-contact and other surfaces easily susceptible to spreading bacterial-related EIDs. Such a surface could be one tool among many others currently in development to mitigate future pandemics caused by bacterial pathogens.

## Supplementary Information

**Additional file 1. ** Supplementary information.

## Data Availability

Not applicable.
