# Supplementary material for: A scalable approach to topographically mediated antimicrobial surfaces based on diamond
Source: J Nanobiotechnology. 2021 Dec 28;19:458. doi: 10.1186/s12951-021-01218-3 (PMC8713538; doi:10.1186/s12951-021-01218-3)
Supplement: Supplementary file 1 — Additional file 1. Supplementary information. [file 12951_2021_1218_MOESM1_ESM.docx]

**A scalable approach to topographically mediated antimicrobial surfaces based on diamond**

**William F. Paxton,*^a^ Jesse L. Rozsa, ^b^ Morgan M. Brooks,^c^ Mark P. Running,^b^ David J. Schultz,^b^ Jacek B. Jasinski,^a^ Hyun Jin Jung, ^b^ and Muhammad Zain Akram*^d^**

*^a^Conn Center for Renewable Energy Research, University of Louisville, Louisville, KY 40292*

*^b^219 Life Sciences Building, University of Louisville, Louisville, KY 40292*

*^c^LSU School of Medicine, 1542 Tulane Ave, New Orleans, LA 70112*

*^d^Kentucky Advanced Materials Manufacturing, Louisville, KY 40209*

**(Additional file 1)**

**Diamond synthesis:**

The diamond nanospike (DNS) structures were synthesized on ~6 cm^2^, 100-oriented silicon substrates via Microwave Plasma-enhanced Chemical Vapour Deposition (MPCVD). Prior to deposition, the silicon substrates were prepared first by a physical abrasion of the surface with 1/10µm metal bond diamond paste (Warren Superabrasives). The substrates were then cleaned and ultrasonicated in a methanol-nanodiamond slurry (Sigma Aldrich 636428) Substrates were cleaned a final time in methanol and placed in a deposition reactor. Diamond nanospike synthesis was performed in an ASTeX microwave plasma enhanced chemical vapor deposition system operating at 2.45 GHz in a hydrogen, methane, and nitrogen environment with gas compositions of 82%, 9%, and 9%, respectively. Power was maintained between 1-1.3 kW, the temperature of substrate holder was set to 850 ^o^C, and pressure was maintained between 40-50 Torr depending on plasma appearance.

The polycrystalline diamond with large grains used for comparison was provided by Kentucky Advanced Materials Manufacturing Co. This was a byproduct of their single crystalline diamond fabrication process. It was grown in a predominantly hydrogen and methane environment.

Atomic force microscopy (AFM) analysis:


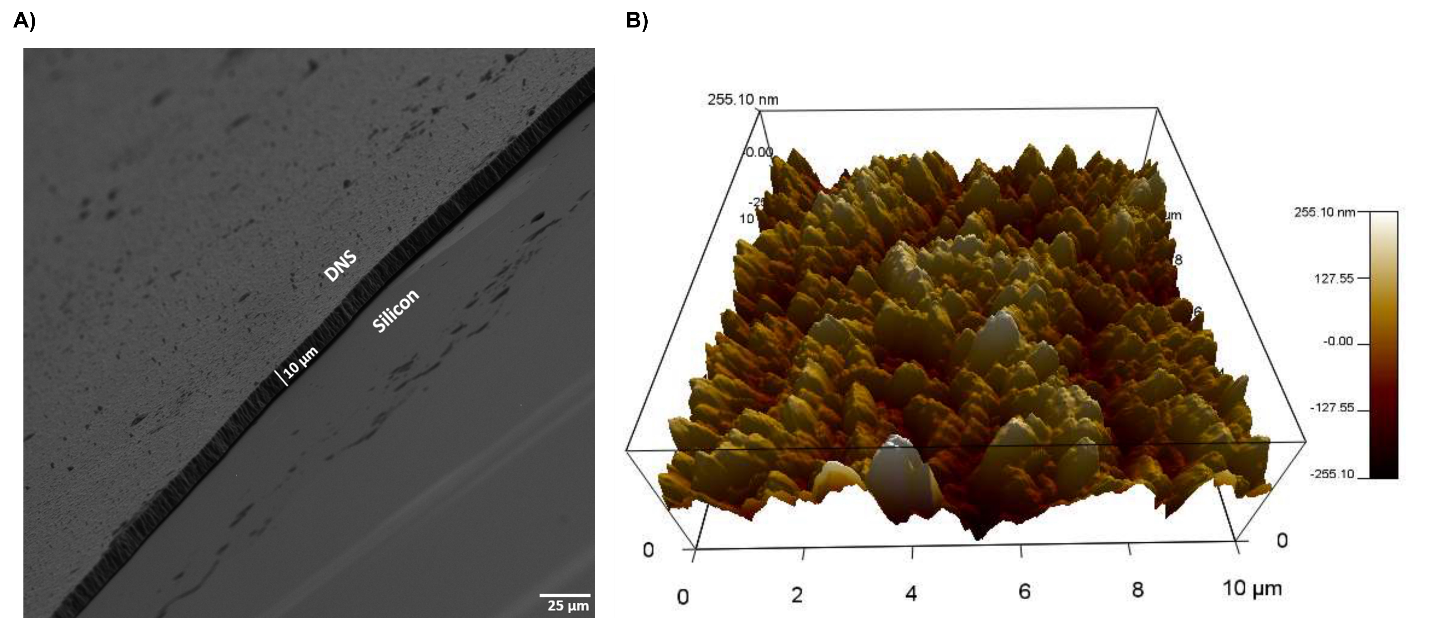


***Figure S1.*** *A) AFM image of DNS film. B) Cross-sectional SEM image of DNS film on silicon substrate*

Figure S1 (A) presents cross-sectional SEM image of tested DNS film. A film thickness of 10 um corresponds to a growth of ~1um/hr. Figure S1 (B) presents AFM image DNS film surface with a root mean square (RMS) surface roughness value of 63.899 nm.

**Raman Analysis:**

Raman measurement was carried out at room temperature using a Horiba LabRam HR800 Confocal System, with 1.5 cm^-1^ resolution, capable of performing Raman in UV to NIR (200 nm -1100 nm) region. CW laser operating at 532.14 nm with a power of 30 mW was utilized obtain the Raman spectra.


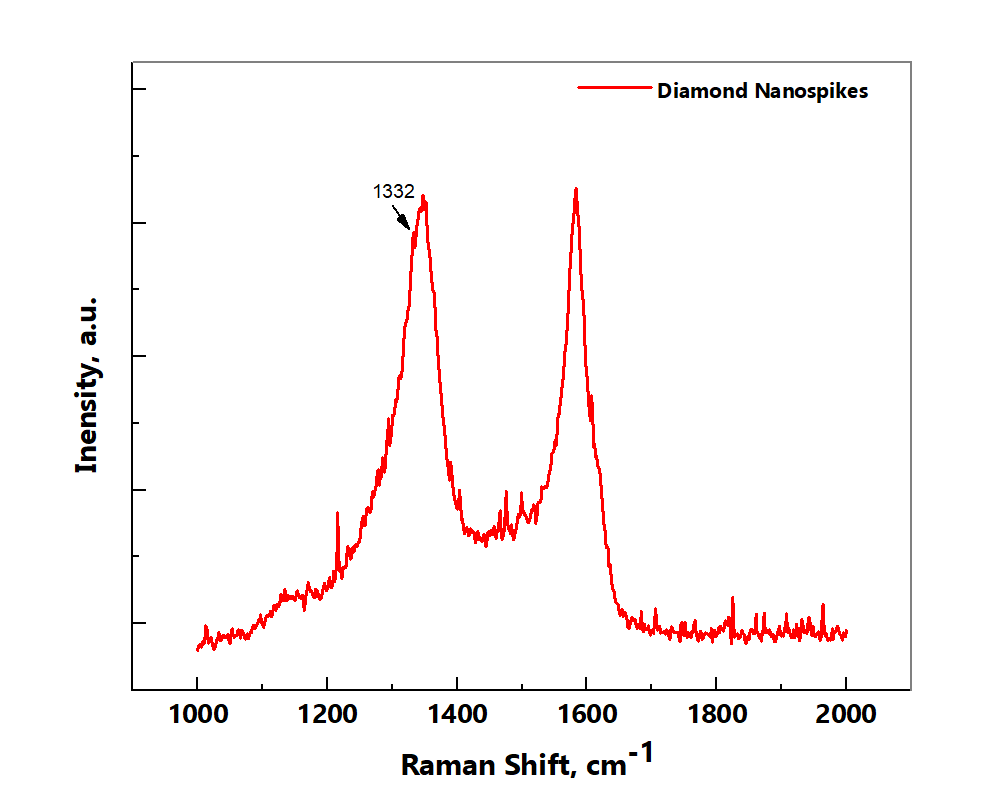


***Figure S2.*** *Raman Spectrum of diamond nanospikes film.*

Raman spectroscopy is a standard tool for identification of diamond phase. Generally, CVD diamond films are comprised of small diamond microcrystals that are usually surrounded by an amorphous carbon phase at grain boundaries. Therefore, in polycrystalline CVD diamond’s Raman spectra, one can observe the Raman peaks for both sp^3^ (~1332 cm^-1^) and sp^2^ (~1580 cm^-1^) hybridizations of carbon (see Figure S1). Noting that Raman spectroscopy is much more sensitive to graphite than diamond, it can be seen that the films are primarily diamond (sp^3^) with a residue of sp^2^ contribution.**[1]**

**SEM characterization:**

The morphology of as-deposited DNS samples and *E. coli* cells disrupted by nanospikes samples was characterized using scanning electron microscopes (TESCAN VEGA3 SBEASYPROBE and Nova NanoSEM 600 FEI) with an acceleration voltage of 15-20 kV.

.


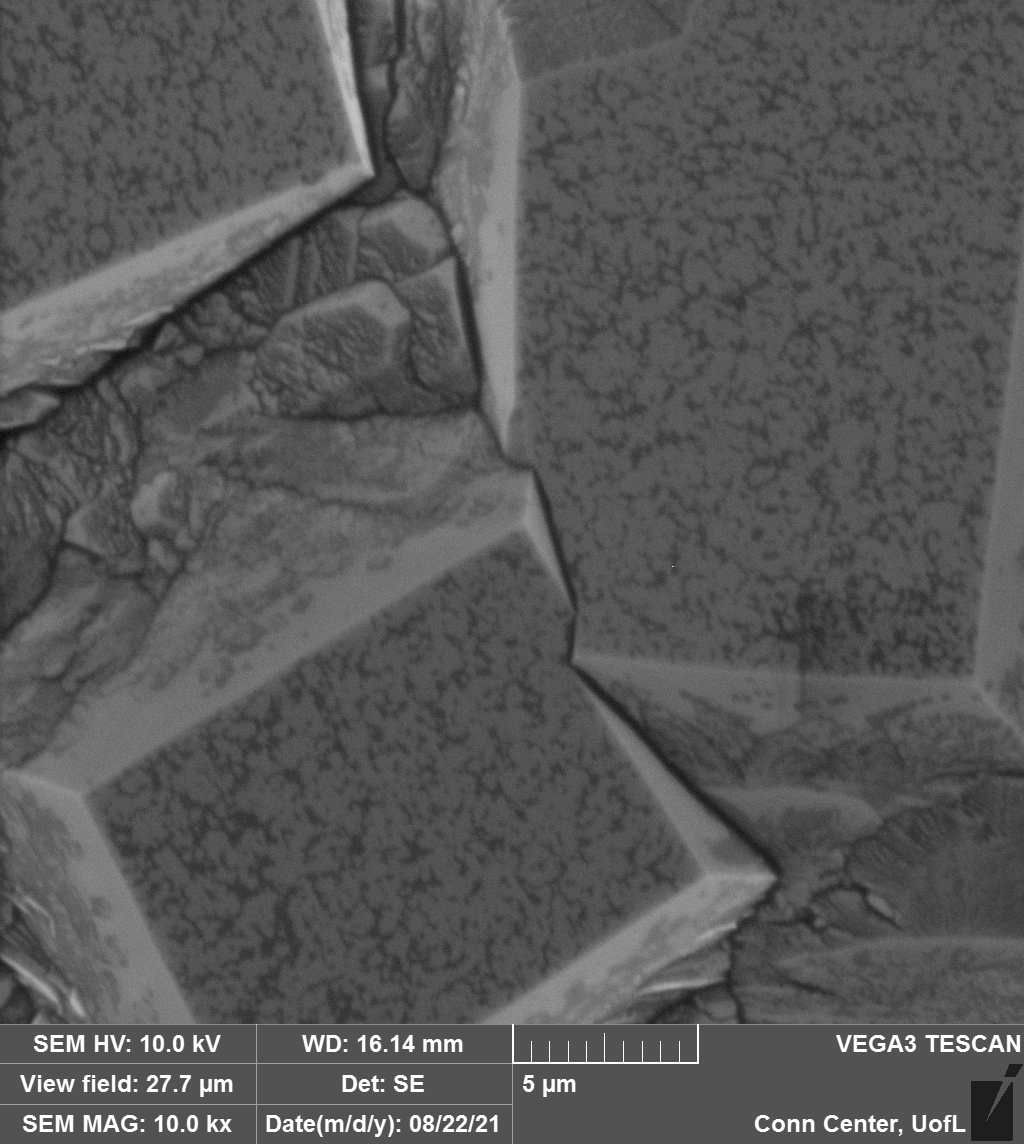

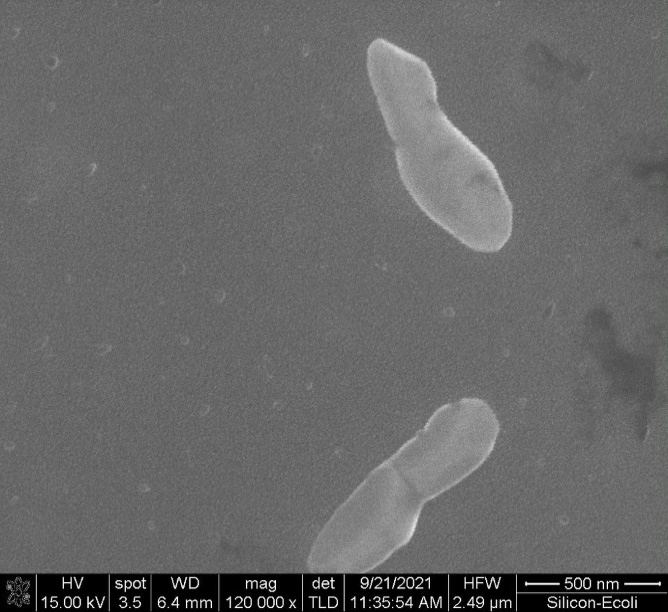


A)

B)

**Figure S3:** *A) Microcrystalline Diamond B) SEM micrograph of E. coli bacteria on silicon surface*

Figure S3 (A) illustrates SEM image of polycrystalline diamond surface with micrometer sized grains used in this study for comparison with diamond nanospike films grown on silicon surface.


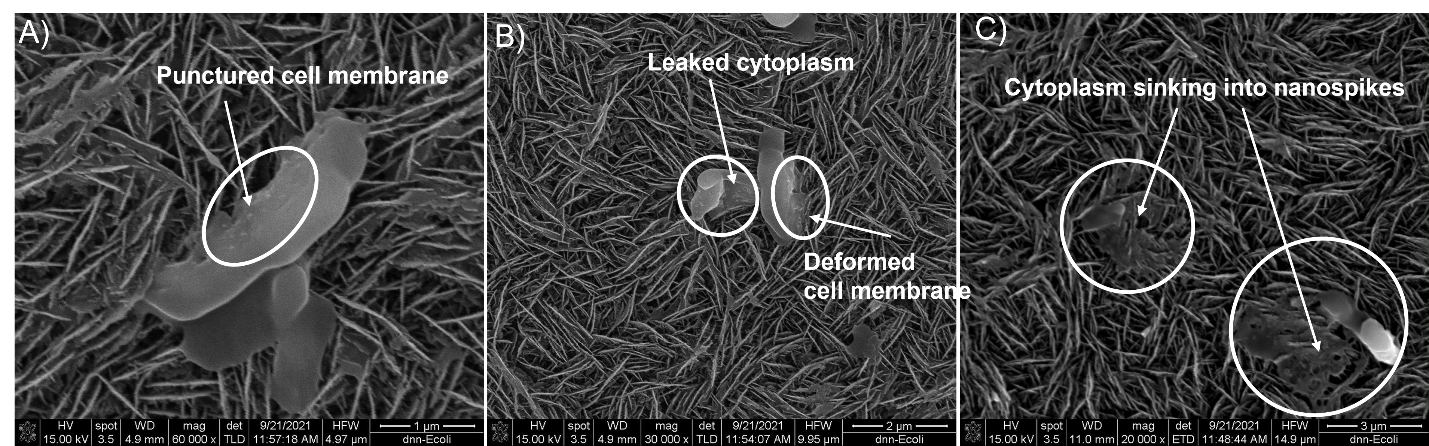


**Figure S4:** *Different levels of E Coli bacteria destruction upon contact with diamond nanospikes.*

The SEM micrographs in Figure S3 (B) and S4 the dead *E. coli* bacteria on silicon and DNS film, respectively. Figure S1 depicts how dead *E. coli* bacteria appears in its intact form on silicon surface. Different levels of *E. coli* cell destruction upon mechanical interaction with diamond nanospikes can be witnessed from Figure S4 (A-C). Figure S4 (A) highlights how the *E. coli* cell still retains its rod-like morphology yet its membrane has been deformed. We can witness the punctured spots, as marked by the arrow. At this point, the cytoplasm begins to leak. Figure S4 (B) highlights the membrane of *E. coli* at a point where most of its cytoplasm is leaked and the cytoplasm begins to get crushed, lying flat on the nanospikes. Figure S4 (C) demonstrates the complete destruction of bacteria after the cytoplasm is fully leaked and merged into the diamond nanospikes. These images are in well agreement with the previously reported topographically mediated surface models.**[2]**

**Qualitative Testing:**

Qualitative testing to observe anti-microbial activity of the diamond nanospike surface as compared to five common materials found on commercially available surfaces (316 stainless steel, galvanized steel, polyethylene plastic, and copper) was performed as follows. Each surface, including a negative control, first had a collection obtained on Day 0 of the experiment to show that none were pre-contaminated with a microorganism prior to the experiment. A collection references the use of a sterile cotton swab dampened with saline to pick up microorganisms from a surface. Collections were then spread across Lysogeny Broth (LB) agar plates. Pre-poured LB agar 100 mm polystyrene plates and sterile 6-inch cotton swabs were purchased from EZ BioResearch LLC. placed in a room temperature (~21-24 ^o^C) environment of standard atmosphere to culture for five days. Pictures were taken on Day 5 (120 hrs) using an AmScope MU1000-CK 10MP USB2.0 Microscope Digital Camera attached to an AmScope binocular compound microscope. On Day 0, after all pre-contaminated collections were obtained, each surface and positive control were exposed to a known bacterial (i.e. positive) source. The known positive source was a collection from the nasal passage and skin of a healthcare worker after a shift on a general medical ward in a large public hospital (University Medical Center New Orleans, LA). Positive exposure utilized individual cotton swabs for each surface to ensure no cross contamination. The negative control was not contaminated with the known positive source. The surfaces were placed in a room temperature (~21- 24 ^o^C) environment of standard atmosphere. Post-exposure collections were taken 48 hrs (Day 2) and 96 hrs (Day 4) after exposure to measure short term anti-microorganismal affect. Collections were spread across LB agar plates that were cultured for five days in the environment described above in the same manner as described for Day 0 above. Pictures were taken on Day 5 of culturing. Figure 2 is the composite of all agar plates cultured for five days after a collection was obtained at Day 0, Day 2, and Day 4.

No growth was observed on the negative control indicating no pre-contamination of any of the samples. The positive control along with 316 stainless steel, galvanized steel, and polyethylene plastic all demonstrated bacterial growth. Copper had only observable fungal growth and Sample A had no growth. This data demonstrates the superior anti-microbial activity of Sample A as compared to other commercially used surfaces including copper which is limited to anti-bacterial activity solely.

As for the collections done 28 days post-exposure to the known positive source, a single collection for each surface was obtained using the same technique as above. This roughly one-month time was chosen as any viable microorganisms should certainly demonstrate some growth by then. The collections were again spread across LB agar plates using sterile cotton swabs (EZ BioResearch LLC) and placed in a room temperature (~21-24 ^o^C) environment of standard atmosphere. Pictures were then taken at the time of collection (0 hrs) and every 24 hrs thereafter for a maximum of 120 hrs. Pictures were taken using a Plugable USB 2.0 Digital Microscope (Plugable Technologies).

Figure S4 shows the negative control had no growth indicating that the cotton swabs, saline, and agar plates used for collections had not been pre-contaminated with any microorganisms. The positive control had growth indicating that the known positive source does contain bacteria. The copper, plastic, and steel samples all had either bacterial growth, fungal growth, or both. Sample A had no growth indicating that it can retain anti-microbial properties for a prolonged time. Interestingly, the copper surface had only fungal growth (consistent with anti-bacterial properties of copper) whereas Sample A had neither bacterial nor fungal growth demonstrating its superior anti-microorganismal properties.


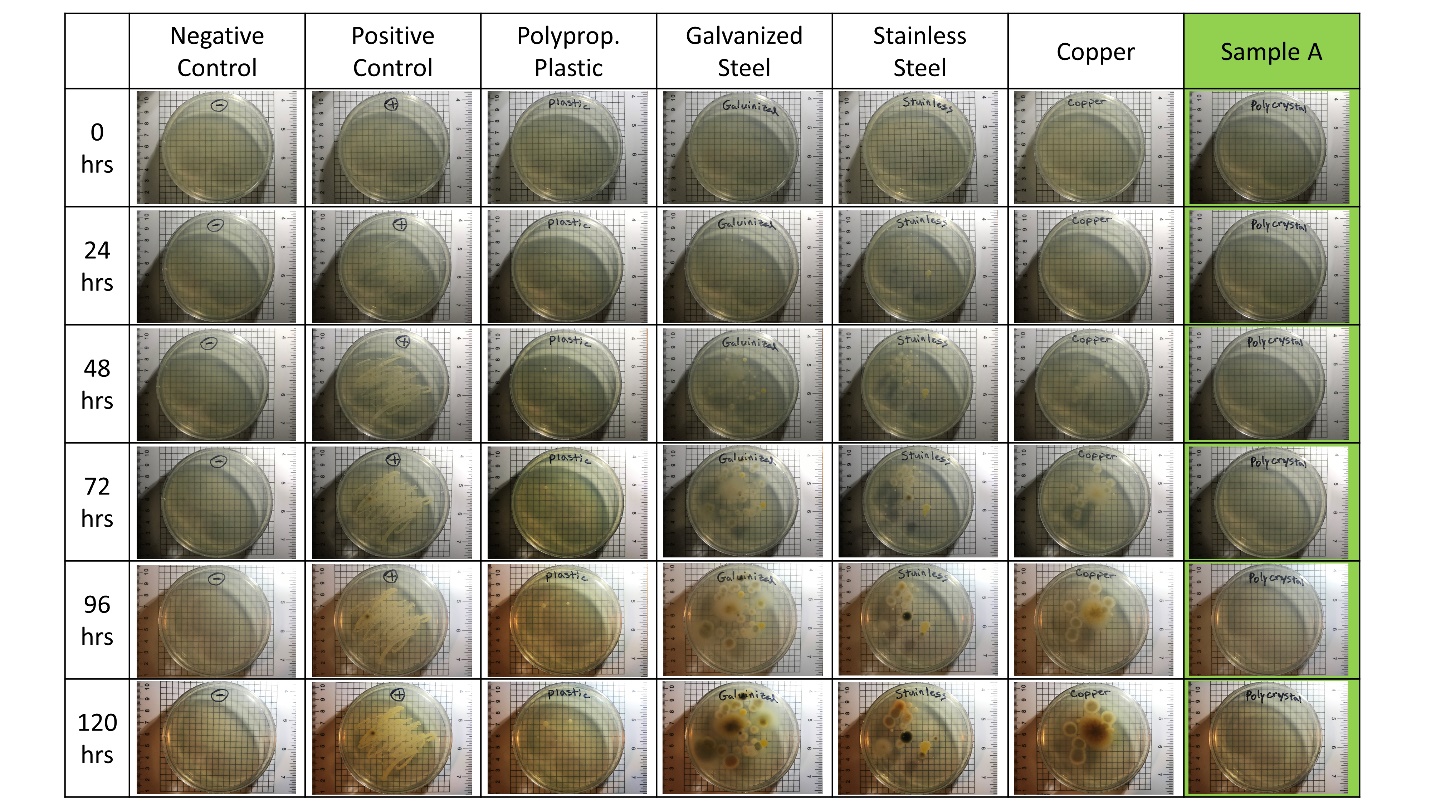


**Figure S5**: *Results of a single collection from each surface taken after 28 days of incubation. Each collection was imaged at 0, 24, 48, 72, 96, and 120 hours. Growth of microorganisms is clearly observable on all surfaces except Sample A.*

**Quantitative and SEM Testing:**

Lysogeny broth (LB) for *E. coli* growth consisted of 10g of Bacto tryptone (Gibco, Life Technologies Corporation), 10 g of sodium chloride (VWR Chemicals), 5 g of yeast extract (Criterion), and mixed in with 1 liter of de-ionized water. The solution then had 15 g of Bacto Agar (Gibco, Life Technologies Corporation) added and the mixture was autoclaved for 20 mins on liquid cycle. After autoclave the liquid mixture was allowed to cool to touch and poured into 100 mm polystyrene cell culture plates (Fischer Scientific). Plates were then allowed to cool to solidification in a sterile hood.

*E. coli* (Thermo-Fischer) was grown in 15 mL of LB liquid media at 37 ^o^C for a 16 hour period. 10 µL of the grown *E. coli* was then placed on each surface (with the exception of negative control), dried in a laminar flow hood for ~15 min, and then placed in a 37 ^o^C incubator for 24 hours. Collections from each surface were taken with 1 mL of sterile LB media and placed into a Microplate, 96 well (Greiner Bio-One) optical density plate. Optical density measurements of *E. coli* were taken at 600 nm every 30 minutes during growth in the spectrophotometer for 14 hours. Spectrophotometer (SpectraMax M2, Molecular Devices) for 14 hours. The software used was SoftMax Pro 7.0.

For SEM measurements, the same process as above was used except the *E.coli*-exposed samples were dried after the 24 hours, 37 ^o^C incubation period. Samples were sputtered with gold and imaged using an FEI Nova 600 SEM.

1. Wada N, Solin SA: **Raman efficiency measurements of graphite.** *Physica B+C* 1981, **105:**353-356.

2. Bandara CD, Singh S, Afara IO, Wolff A, Tesfamichael T, Ostrikov K, Oloyede A: **Bactericidal Effects of Natural Nanotopography of Dragonfly Wing on Escherichia coli.** *ACS Applied Materials & Interfaces* 2017, **9:**6746-6760.
